# Neutralizing misinformation through inoculation: Exposing misleading argumentation techniques reduces their influence

**DOI:** 10.1371/journal.pone.0175799

**Published:** 2017-05-05

**Authors:** John Cook, Stephan Lewandowsky, Ullrich K. H. Ecker

**Affiliations:** 1 Center for Climate Change Communication, George Mason University, Fairfax, Virginia, United States of America; 2 School of Psychological Science, University of Western Australia, Perth, Western Australia, Australia; 3 School of Experimental Psychology and Cabot Institute, University of Bristol, Bristol, United Kingdom; Kyoto University, JAPAN

## Abstract

Misinformation can undermine a well-functioning democracy. For example, public misconceptions about climate change can lead to lowered acceptance of the reality of climate change and lowered support for mitigation policies. This study experimentally explored the impact of misinformation about climate change and tested several pre-emptive interventions designed to reduce the influence of misinformation. We found that false-balance media coverage (giving contrarian views equal voice with climate scientists) lowered perceived consensus overall, although the effect was greater among free-market supporters. Likewise, misinformation that confuses people about the level of scientific agreement regarding anthropogenic global warming (AGW) had a polarizing effect, with free-market supporters reducing their acceptance of AGW and those with low free-market support increasing their acceptance of AGW. However, we found that inoculating messages that (1) explain the flawed argumentation technique used in the misinformation or that (2) highlight the scientific consensus on climate change were effective in neutralizing those adverse effects of misinformation. We recommend that climate communication messages should take into account ways in which scientific content can be distorted, and include pre-emptive inoculation messages.

## Introduction

Misinformation, that is, information that people accept as true despite it being false, can have significant societal consequences. For example, denial of the scientific consensus that HIV causes AIDS led to policies in South Africa between 2000 and 2005 that are estimated to have contributed to 330,000 excess deaths [1]. In Western countries, decreased acceptance of vaccinations based on erroneous or exaggerated claims of adverse effects has led to lower compliance; this has placed the population at greater risk of vaccine-preventable disease [2,3,4], and likely led to the U.S. measles outbreaks in 2014 and 2015 [5,6].

Given the plethora of information that confronts individuals every day, it should come as no surprise that people do not and cannot assess every piece of information on its merit. Rather, heuristics—mental rules-of-thumb—are frequently applied when evaluating claims and evidence: Have I heard this before? Does it fit in with what I already know? What do relevant others think about it? As with all heuristics, this can be an effective strategy in many circumstances [7], but it is prone to bias, especially when particular myths are frequently encountered, when existing knowledge is incorrect, and/or when one’s social neighborhood shares or even identifies through false beliefs. Individuals do not seek and interpret information in a neutral, objective manner—rather, people tend to favor information that confirms existing beliefs [8]. Arguably, this confirmation bias is particularly strong when the underlying belief or attitude is also particularly strong, in which case counter-attitudinal evidence is frequently dismissed uncritically.

## The effects of worldviews on the acceptance of evidence

The behavioral and societal consequences of misinformation underscore the need to improve our understanding of how misinformation might be corrected and its influence reduced. However, this can be a problematic exercise because misperceptions have been found to be remarkably persistent to corrections, and interventions are known to backfire if applied incorrectly. Perhaps the most pervasive backfire effect involves information that challenges people’s “worldviews”; that is, their fundamental beliefs about how society should operate. The worldview backfire effect refers to the fact that when corrective evidence contradicts a person’s prior beliefs, their beliefs may ironically be strengthened despite the evidence [9,10]. For example, in one study, conservatives became *more* likely to believe that Iraq had weapons of mass destruction (WMDs) immediately before the war of 2003 after reading retractions clarifying that no WMDs existed at the time [11]. Similarly, receiving information about the scientific consensus on anthropogenic global warming (AGW) can cause participants with strong support for free, unregulated markets to become *less* accepting of climate change [12].

As misinformation is often resistant to correction—in particular if a correction challenges a person’s worldview—alternative avenues of dampening the impact of misinformation need to be explored. One promising approach, derived from inoculation theory [13,14], is to prepare people for potential misinformation by exposing some of the logical fallacies inherent in misleading communications *a priori*. The rationale of this pre-exposure is that by “inoculating” people in this manner, they will subsequently recognize flawed arguments and dismiss them as deceptive.

To foreshadow briefly, in two experiments we looked at two sides of the misinformation coin: we examined the effects of misinformation on climate attitudes, and we sought to eliminate the effects of that misinformation through the exploration of various types of counter-information provided *before* exposure to the misinformation. We were particularly interested in whether our counter-information approach would be able to offset misinformation effects even when the counter-information conflicted with people’s worldview and might therefore be received critically. In both experiments, the manipulations related to the scientific consensus on climate change, focusing on misleading strategies that aim to undermine the perceived consensus either through demonstrating a “false balance” of evidence (Experiment 1) or by presenting evidence from “fake experts” (Experiment 2). In the following, we first elaborate on the general effects of worldview on the acceptance of evidence, before we address the scientific consensus on climate change, and review the literature on inoculation theory.

In general, evidence is often rejected if it threatens a person’s worldview. In the case of climate science, the worldview that is threatened by the bulk of the scientific evidence is political conservatism. Accepting the evidence that human activities drive climate change suggests embracing behavioral change, including support of increased regulation of free markets. This sits uncomfortably with conservative values of liberty and freedom. Accordingly, climate change perceptions and attitudes have repeatedly been found to be strongly associated with political worldview [15,16,17,18].

Trust in climate scientists also plays a part in shaping climate attitudes [19]. Rejection of climate change has been associated with conspiratorial thinking [17,20], and conspiratorial images are the most common reaction to climate change information amongst those who reject climate science [21]. Recently, a cognitive model based on Bayesian belief networks found that the potentially conspiratorial trait of ‘active distrust of scientists’ was a key component of the cognitive processes leading to the rejection of evidence among a small proportion of participants with strong support for free, unregulated markets [12].

In sum, worldview can lead people to embrace misinformation without scrutiny, and (as reviewed earlier) to also dismiss counter-attitudinal corrections. Indeed, worldview-dissonant corrections can even backfire and further entrench misconceptions. Worldview also influences perception of scientific consensus on climate change, as well as how people respond to information about consensus.

## Distortions of scientific consensus

Several studies have found nearly unanimous agreement among publishing climate scientists that humans are causing global warming [22,23,24], and a similar pervasive consensus exists in the scientific literature [25,26]. A frequently-cited figure puts the consensus at around 97% of publishing scientists and of relevant peer-reviewed articles. However, among the general public, the *perception* of this scientific consensus is considerably lower, and hovers around 57–67% across recent studies [27]. This gap between public perception and the 97% level of actual agreement is significant because perceived consensus has been identified as a “gateway belief” that influences a number of other attitudes about climate change and climate solutions [28,29,30].

One reason why the public may be generally under-estimating the consensus is because of the prominence of political operatives and lobbyists who dissent from the consensus in public discourse. Those individuals appear to have relevant expertise but in fact rarely do (i.e., they are ‘fake experts’) [31]. An early example of this strategy was the 1995 “Leipzig Declaration”, a document purporting to refute the scientific consensus on climate change. However, among the 105 signatories, many worked in fields unrelated to climate, and 12 even denied signing the document altogether [32]. Texts featuring fake experts that cast doubt on the consensus have been observed to lower perceived consensus and acceptance of climate change [33].

Another potential contributor to low perceived consensus is media coverage that evenly balances contrarian voices and expert views (i.e. ‘false balance’ coverage). Media coverage of scientific issues has diverged from the scientific consensus on issues such as climate change [34,35,36] and the mythical vaccine-autism link [37]. False-balance media coverage has been observed to decrease public certainty about scientific issues when it comes to environmental science [38], the false link between vaccination and autism [39], and the health effects of pollution [40]. The presence of potentially credible fake experts and the false balance presented by the media necessitates that communicators effectively reduce the influence of misinformation.

## Prebunking and inoculation theory

Given the difficulties associated with correcting misinformation once it has been processed [10], an alternative approach is to neutralize potential misinformation *before* it is encoded, a technique colloquially known as “prebunking” [41]. In a field study involving pre-existing attitudes, it was found that people who were suspicious of the U.S. government’s motives for the invasion of Iraq in 2003 were subsequently less likely to believe in retracted misinformation—information that had been explicitly identified as false after initially being judged true—about the war [42]. In other research, pre-existing reputations of a company were observed to influence how corporate philanthropic messages are received, with a bad reputation resulting in corporate charitable behavior being seen as a self-interested activity [43].

These studies indicate that pre-existing attitudes influence how people respond to new information (or misinformation). Similarly, inoculation theory proposes that people can be “inoculated” against misinformation by being exposed to a refuted version of the message beforehand [14]. Just as vaccines generate antibodies to resist future viruses, inoculation messages equip people with counterarguments that potentially convey resistance to future misinformation, even if the misinformation is congruent with pre-existing attitudes.

There are two elements to an inoculation: (1) an explicit warning of an impending threat and (2) a refutation of an anticipated argument that exposes the imminent fallacy. For example, an inoculation might include (1) a warning that there exist attempts to cast doubt on the scientific consensus regarding climate change, and (2) an explanation that one technique employed is the rhetorical use of a large group of “fake experts” to feign a lack of consensus. By exposing the fallacy, the misinformation (in this case, the feigned lack of consensus) is delivered in a “weakened” form. Thus, when people subsequently encounter a deceptive argument, the inoculation provides them with a counter-argument to immediately dismiss the misinformation.

Inoculation messages have been found to be more effective at conveying resistance to misinformation than supportive messages (i.e., messages that promote accurate information without mentioning the misinformation) [44]. Inoculation messages are also useful in behavior-change interventions, with participants responding positively (compared to a control group) to inoculations against arguments justifying alcohol consumption [45], the threat of peer-pressure leading to smoking initiation [46], and pro-sugar arguments from soda companies [47]. Inoculation can reduce the influence of conspiracy theories by increasing the degree of scepticism towards conspiratorial claims [48], and has been shown to convey resistance to misinformation regarding agricultural biotechnology [49]. Inoculation is effective with people possessing different pre-existing attitudes—a situation particularly relevant to the climate change issue [49]. Also of relevance, given that individualism and free-market support are strong drivers of climate attitudes, is the fact that emphasizing the dubious practices of an information source can shed light on how misinformation impinges on people’s freedom to be accurately informed, thus potentially enhancing the effectiveness of inoculations among conservatives [50].

Inoculation has been tested experimentally in the context of climate change. When participants were exposed to both consensus information and misinformation casting doubt on the consensus, there was no significant change in acceptance of climate change [33]. This result indicates that the positive effect of accurate information can be potentially undone by misinformation. The study also found that a significant increase in AGW acceptance occurred when the consensus information was coupled with an inoculation explaining the technique employed by misinformers, prior to receiving the misinformation.

This article addresses two questions left open by previous research. First, what effect does misinformation have on acceptance of climate change? Second, can inoculation neutralize the influence of misinformation? We examined several ways of inoculating against climate-change-related misinformation, by explaining the techniques used to sow doubt about the science. We also extended van der Linden et al.’s (2017) [33] study by exploring the impact of inoculation on two types of misinformation: arguments that *implicitly* cast doubt on the consensus using ‘false-balance’ coverage, and arguments that *explicitly* cast doubt on the consensus. Experiment 1 looked at misinformation in the form of ‘false balance’ media coverage, which misinforms by conveying the impression of evenly balanced discourse in the scientific community regarding climate change. Experiment 2 looked at explicit misinformation that seeks to manufacture doubt about the scientific consensus by employing the ‘fake experts’ strategy. In both studies, the effectiveness of inoculations was compared to conditions in which misinforming messages were left uncorrected.

## Experiment 1

### Method

Experiment 1 tested the effect of inoculation against misinformation that takes the form of ‘false balance’ media coverage regarding climate change. Specifically, we used a news article that presented mainstream scientific views alongside contrarian scientists’ views. False-balance media coverage of this type has been shown to confuse the public on various scientific topics [39,51,52,40]. Two types of information were shown prior to the misinformation: (1) consensus information, which has been shown to significantly increase acceptance of climate change in the vast majority of recipients [29,12,30], and/or (2) an inoculation explaining the misleading effects of false-balance media coverage. As the purpose of the experiment was to determine the efficacy of inoculation *before* exposure to misinformation, consensus information was also shown prior to the misinformation in order to observe its relative efficacy in comparison to the inoculation intervention.

The misinformation text was a mock news article that first featured scientists presenting research supporting AGW, followed by contrarian scientists rejecting AGW and proposing alternative explanations (S1). The consensus information was a text-only description of various studies reporting 97% scientific agreement on human-caused global warming. The inoculation information was a textual explanation of the “false balance” strategy used by the tobacco industry to confuse the public about the level of scientific agreement by staging a fake debate.

Participants were randomly assigned to one of five groups: a control group and four groups who were presented with misinformation. For the four misinformation groups, consensus information and inoculation information were fully crossed so that prior to the misinformation, participants either read consensus information, inoculation information, a message combining both consensus and inoculation information, or no message. The latter condition differed from the control group only in that it contained misinformation. The study was approved by the Human Research Ethics Committee at the University of Western Australia, with participants indicating consent through participation in the online survey.

### Participants

Participants (*N* = 1092) were a U.S. representative sample recruited through Qualtrics.com, selected by gender, age, and income demographics that we provided (49.0% female, average age *M* = 48 years, *SD* = 15 years)—a procedure which has been shown to ensure reasonably approximate representativeness [53]. Qualtrics delivered 751 completes (after eliminating 341 participants who failed attention filters; see below for details) that comprised the initial sample for analysis. We then eliminated 30 participants who entered null perceived consensus (*n* = 18), null age (*n* = 2), age greater than 100 (*n* = 2) or who took excessive time to complete the survey (*n* = 15). The time threshold to complete the survey was calculated according to the outlier labeling rule (square-root transformed duration more than 2.2 times the inter-quartile range above the 3^rd^ quartile) [54]. The final sample of participants (*N* = 714) were randomly allocated to one of five groups: Control (*n* = 142), Misinformation (*n* = 145), Consensus/Misinformation (*n* = 142), Inoculation/Misinformation (*n* = 142) and Consensus/Inoculation/Misinformation (*n* = 143).

### Test items

The survey included 37 survey items (S1 Table). In addition, to ensure attentive reading of the intervention text, the survey included two generic attention filters plus an additional attention filter for groups that included the misinformation intervention. Only the 751 participants who filled out all survey items, including correct entry of attention-filter questions, were included in the sample and forwarded to the authors by Qualtrics.

The survey items measured seven constructs: AGW acceptance, free-market support, trust in climate scientists, trust in contrarian scientists, attribution of long-term climate trends to human activity (henceforth “attribution”), perceived consensus, and mitigative climate policy support (henceforth “policy support”). AGW acceptance was measured using five items from [29]. Free-market support was used as a proxy for political ideology, given the strong relationship between free-market support and climate attitudes [15]. While there is a strong correlation between conservatism and free-market support, there are also nuanced distinctions between these two measures. For example, Lewandowsky, Gignac, and Oberauer [20] found that free-market support was negatively associated with vaccine support while conservatism was positively associated with vaccine support. Nevertheless, Lewandowsky, Gignac, and Oberauer also found that both free-market support and conservatism were negatively associated with climate attitudes; thus, we consider free-market support an appropriate proxy for political ideology. Perceived consensus was assessed on a slider from 0 to 100%. Attribution was measured using three scales (ranging from 0 to 100%) estimating the human contribution to temperature change, sea level rise, and extreme weather events. Policy support was measured with 5 items adapted from Ding et al. [28].

Five items measuring trust in climate scientists were adapted from Ohanian [55]. Trust in contrarian scientists was measured because the interventions referred to contrarian scientists who cast doubt on the scientific consensus on human-caused global warming. The five items measuring trust in contrarian scientists were adapted from the trust in climate scientists items. For example, “Climate scientists can be depended upon to help increase our understanding of what’s happening to our climate” was changed to “Scientists who reject the scientific consensus on global warming can be depended upon to increase our understanding of what’s happening to our climate”.

### Results

For the analyses, responses were averaged across items for each construct where applicable. In our analysis, we first ascertained whether there was an effect of the misinformation intervention. We next focused on the two-way interaction between the consensus and inoculation interventions for the four groups that received misinformation. The dependent variable of greatest interest was perceived consensus, given its status as a gateway belief [30] and the fact that the misinformation interventions focused on the concept of consensus. Table 1 summarizes the means and standard deviations of the dependent variables for each group.

**Table 1:** Means (standard deviations) across interventions for experiment 1.

Dependent Variable	Control	Misinformation- only	Consensus + Misinformation	Inoculation + Misinformation	Consensus + Inoculation + Misinformatiion
Perceived consensus	68.9 (22.5)	63.5 (21.8)	86.1 (18.1)	70.0 (27.9)	83.9 (22.4)
AGW acceptance	3.40 (.86)	3.25 (.94)	3.52 (.87)	3.46 (.90)	3.53 (.93)
Attribution	50.7 (27.0)	47.0 (26.7)	53.4 (28.0)	53.2 (28.4)	54.4 (26.3)
Trust in climate scientists	3.35 (.88)	3.26 (.82)	3.47 (.82)	3.28 (.73)	3.44 (.86)
Trust in contrarian scientists	3.34 (.60)	3.38 (.73)	3.46 (.56)	3.20 (.74)	3.27 (.75)
Policy support	3.55 (.89)	3.50 (.92)	3.70 (.79)	3.53 (.86)	3.61 (.91)

### Effect of misinformation

A *t*-test was conducted to compare perceived consensus in the control condition versus the condition that received misinformation only, finding a significant difference; *t*(284) = 2.05, *p* = .046. This indicates that misinformation in the form of false-balance media articles has a negative effect on public perception of scientific consensus. The effect of misinformation failed to reach statistical significance for the other dependent variables.

### Effect of interventions preceding the misinformation

The next stage of our analysis determined the effect of consensus information and inoculation presented prior to the misinformation by focusing on the four groups that received misinformation (i.e., excluding the control group). For these four groups, separate Type II ANOVAs were performed using the Car package for the R statistical programming environment for the six dependent measures (perceived consensus, AGW acceptance, attribution, trust in climate scientists, trust in contrarian scientists, and policy support) with the consensus and inoculation interventions as fully-crossed factors. Free-market support was included as an additional continuous predictor. Table 2 summarizes the ANOVA results.

**Table 2:** ANOVA results for Experiment 1.

Dependent Variable	Effects	η_p_^2^	F	p
Perceived consensus	Consensus	.003	89.270	< .001***
	Inoculation	.001	.723	.395
	Free-Market Support	.038	24.378	< .001***
	Consensus × Inoculation	.000	4.595	.033*
	Consensus × Free-Market Support	.002	1.191	.276
	Inoculation × Free-Market Support	.001	.371	.543
	Consensus × Inoculation × Free-Market Support	.001	.573	.450
AGW acceptance	Consensus	.002	3.398	.066
	Inoculation	.000	.852	.356
	Free-Market Support	.322	276.911	< .001***
	Consensus × Inoculation	.000	1.189	.276
	Consensus × Free-Market Support	.001	.452	.502
	Inoculation × Free-Market Support	.000	.000	.989
	Consensus × Inoculation × Free-Market Support	.001	.287	.593
Attribution	Consensus	.000	1.562	.212
	Inoculation	.000	1.409	.236
	Free-Market Support	.136	88.288	< .001***
	Consensus × Inoculation	.002	.804	.370
	Consensus × Free-Market Support	.000	.052	.819
	Inoculation × Free-Market Support	.001	.628	.429
	Consensus × Inoculation × Free-Market Support	.001	.613	.434
Trust in climate scientists	Consensus	.014	5.775	.017*
	Inoculation	.000	.421	.516
	Free-Market Support	.181	127.877	< .001***
	Consensus × Inoculation	.000	.021	.885
	Consensus × Free-Market Support	.009	5.226	.023*
	Inoculation × Free-Market Support	.000	.008	.927
	Consensus × Inoculation × Free-Market Support	.000	.251	.617
Trust in contrarian scientists	Consensus	.011	3.122	.078
	Inoculation	.012	8.286	.004**
	Free-Market Support	.149	107.772	< .001***
	Consensus × Inoculation	.005	.143	.705
	Consensus × Free-Market Support	.008	4.187	.041*
	Inoculation × Free-Market Support	.007	3.622	.058
	Consensus × Inoculation × Free-Market Support	.005	2.761	.097
Policy support	Consensus	.011	1.976	.160
	Inoculation	.012	1.444	.230
	Free-Market Support	.149	202.339	< .001***
	Consensus × Inoculation	.005	.372	.542
	Consensus × Free-Market Support	.008	.331	.565
	Inoculation × Free-Market Support	.007	.080	.777
	Consensus × Inoculation × Free-Market Support	.005	2.857	.092

ANOVA is conducted on 4 groups that received misinformation, forming a 2 × 2 fully-crossed design crossing the consensus and inoculation interventions. In the Effects column, Consensus refers to the consensus intervention, Inoculation refers to the inoculation intervention.

* p < .05.

** p < .01.

*** p < .001.

**Fig 1:**
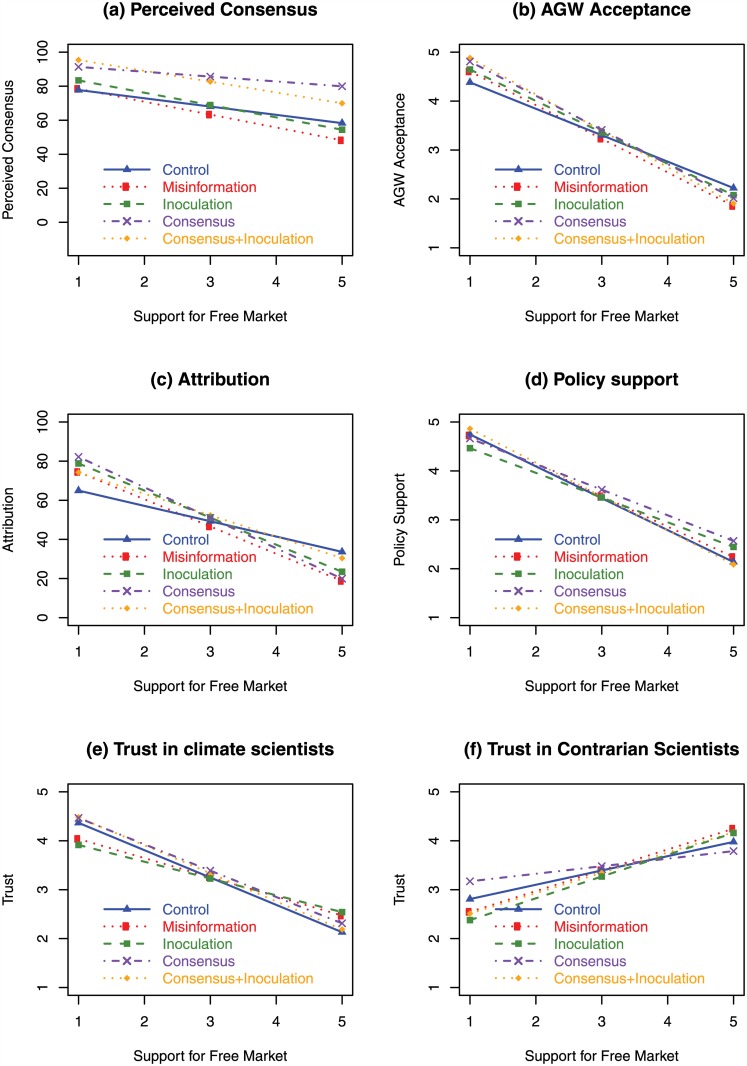
Predicted response in Experiment 1 from linear regression of observed data. Blue solid line with triangles represents control group, red dotted line with circles represents group receiving misinformation only, green dashed line with squares represents group receiving inoculation before misinformation, purple dot-dashed line with crosses represents group receiving consensus information before misinformation, orange dotted line with diamonds represent group receiving consensus plus inoculation information before misinformation. Horizontal axis represents free-market support where 5 corresponds to strong agreement with unregulated markets. (a) Perceived scientific consensus on AGW. (b) AGW acceptance. (c) Attribution of human activity to climate trends. (d) Policy support. (e) Trust in climate scientists. (f) Trust in contrarian scientists.

Fig 1 shows the effect of the different interventions on the six dependent variables. The greatest effects were seen in perceived consensus, shown in Fig 1(a). Compared to the Control group (blue solid line, *M* = 68.9%), the misinformation (red dotted line) decreased perceived consensus (*M* = 63.5%), with the greatest effect on strong free-market supporters. Conversely, presenting consensus information prior to the misinformation nullified the negative influence of the false-balance misinformation by increasing perceived consensus (*M* = 86.1%). The reduced slope of the consensus group (purple dot-dashed line) indicates that the consensus information partially moderated the influence of free-market support. Inoculation (green dashed line) also neutralized the misinformation, with no overall change in perceived consensus (relative to control). Presenting the consensus information along with the inoculation text also caused a significant increase in perceived consensus (*M* = 83.9%), to a similar level as consensus-only.

The interventions had greatest effect on perceived consensus, while other climate attitudes showed a weaker effect, consistent with other studies finding that changes in perceived consensus propagate to other climate attitudes to a lesser degree [30]. Trust in climate scientists, shown in 1(e), was significantly increased by the consensus intervention (relative to the misinformation condition). Fig 1(f) demonstrates the main effect of the inoculation on trust in contrarian scientists, with the inoculation group (green solid line) showing decreased trust in contrarian scientists relative to the control group (blue dashed line). There was also an interaction between the consensus information and free-market support on trust in both climate and contrarian scientists, with trust decreasing among participants with high free-market support.

In sum, the effect of false-balance media coverage had the greatest effect on perceived consensus among the various climate attitudes measured. However, a consensus message presented with the false-balance message was effective in increasing perceived consensus, thus neutralizing the negative influence of the misinformation. In addition, we found that an inoculation message was effective in neutralizing the effect of misinformation on perceived consensus.

### Discussion

Experiment 1 found that misinformation in the form of “false balance” media articles decreased perceived consensus. This result is consistent with McCright, Charters, Dentzman, and Dietz [56], who found that false-balance media articles decreased acceptance of climate change, attitudes towards climate science, awareness of climate change consequences, and support for greenhouse gas emission reductions. McCright et al. also found that climate misinformation was most effective on conservatives, while having no effect on liberals.

Exploring the efficacy of inoculation interventions on perceived consensus, Experiment 1 found that pre-emptively explaining the potentially misleading effect of false-balance media coverage was effective in neutralizing the negative influence of that type of misleading media coverage. While inoculations have been found in this analysis and other studies to be effective in neutralizing misinformation, an open question is the efficacy of positive information that is countered with misinformation. Van der Linden et al. [33] found that the positive effect of consensus information was cancelled out by the presence of misinformation. In contrast, our Experiment 1 found that consensus information was the most effective intervention in conferring resistance to false-balance media coverage. One possible explanation for the conflicting results may be the nature of the misinformation. In [33], the misinformation explicitly cast doubt on the consensus using text from the Oregon Petition Project (similar to our Experiment 2; see below). In contrast, the misinformation in our Experiment 1 *implied* a lack of consensus in a less direct manner, by presenting mainstream science and dissenting viewpoints on an equal footing.

Also of note was that for the group exposed to consensus information, the impact of free-market support on perceived consensus and trust in contrarian scientists was attenuated, indicating a neutralizing influence of consensus information consistent with other studies [29,30]. In a similar vein, Deryugina and Shurchkov [57] found consensus information to have equal impact among liberals, moderates, and conservatives. However, our results conflict with the results of [12], who found that consensus messaging had a polarizing effect on climate attitudes of American respondents—that is, strong free-market supporters responded to a consensus message by reducing acceptance of AGW while liberals responded by increasing acceptance of AGW. It is difficult, therefore, to draw firm conclusions from the available research. It seems that in general, consensus information moderates the influence of ideology, but further research should try to pinpoint boundary conditions under which consensus information may polarize (as found in [12]).

## Experiment 2

### Method

Experiment 2 tested the impact of misinformation that explicitly seeks to manufacture doubt about the scientific consensus on climate change. One way to achieve this is through the use of non-experts to cast doubt on expert agreement, which is known as the “fake experts” strategy [31]. Experiment 2 also tested whether inoculating participants prior to reading misinformation was effective in neutralizing the influence of the misinformation. The experiment thus featured a 2 × 2 between-subjects design, fully crossing a misinformation intervention and an inoculation intervention, such that participants were divided into a control group (no intervention text), inoculation group (inoculation with no misinformation), misinformation group (misinformation with no inoculation), and inoculation/misinformation group (inoculation preceding misinformation). The study was approved by the Human Research Ethics Committee at the University of Western Australia, with participants indicating consent through participation in an online survey.

### Participants

Participants (*N* = 400) were a representative U.S. sample, recruited through Qualtrics.com, based on U.S. demographic data on gender, age, and income in the same fashion as for Experiment 1 (49.2% female, average age *M* ≈ 43 years, *SD* ≈ 15 years). The sample delivered by Qualtrics comprised only participants who had successfully answered all attention filter items. None of the participants had participated in Experiment 1. Outliers in the time taken to complete the survey (*n* = 8) were eliminated according to the outlier labelling rule as in Experiment 1. The final sample of participants (*N* = 392) were randomly allocated to the four experimental conditions: control (*n* = 98), inoculation (*n* = 98), misinformation (*n* = 99), and inoculation+misinformation (*n* = 97).

### Materials

The misinformation intervention consisted of text taken verbatim from the Global Warming Petition Project website, a website run by the so-called Oregon Institute of Science and Medicine. The text mentions a petition of over 31,000 signatories with science degrees who have signed a statement claiming that human release of greenhouse gases is not causing disruption of the Earth’s climate (the so-called “Oregon Petition”). The text argues that because a large number of scientists reject the hypothesis of human-caused global warming, there is no scientific consensus.

This argument is misleading as the minimum qualification required to be a signatory of the Oregon Petition is a Bachelor’s degree in science. The 31,000 signatories comprise only around 0.3% of the 10.6 million U.S. science graduates since the 1970/71 school year [58]. The list contains no affiliations, making verification of signatories problematic. Further, over 99% of the signatories have no expertise in climate science. Despite the use of the “fake expert” strategy, the Oregon Petition is an effective rhetorical argument. Van der Linden [33] found that the Oregon Petition was the most effective among six climate contrarian claims in reducing acceptance of climate change. The misinformation text used here (406 words) consisted of a mixture of text and a screenshot of the signed Oregon Petition.

The inoculation intervention explained the technique of “fake experts”, that is, the use of spokespeople who convey the impression of expertise without possessing any relevant scientific expertise. Specifically, the text used the example of a tobacco industry ad featuring tens of thousands of physicians endorsing a particular brand of cigarette. The inoculation text (358 words) consisted of a mixture of text and a figure of a tobacco ad with the text ‘20,679 Physicians say “Luckies are less irritating” [59]. This ad was used for two reasons: first, because the use of tens of thousands of physicians echoed the large numbers invoked by the Oregon Petition. Second, tobacco was used as an example rather than explicitly mentioning the Oregon Petition, so that participants were inoculated against the general technique of “fake experts” rather than a specific instance of misinformation. Finally, the text compared the tobacco strategy to similar approaches used in climate change, without specifically mentioning the Oregon Petition. Participants exposed to the misinformation intervention were shown debriefing text after completing the survey (S10).

Participants’ post-intervention attitudes were measured via a survey. The survey included 36 items (listed in S2 Table) plus between zero (for the control group with no text interventions) to two attention-filter items—designed to ensure participants were attending to the interventions. The measured constructs in Experiment 2 matched those used in Experiment 1, except that a measure of trust in contrarian scientists was not included. The six measured constructs were thus free-market support, perceived scientific consensus, AGW acceptance, attribution, trust in climate scientists, and policy support. In addition, some items tested people’s views on how others might be affected by the experimental messages. Those were collected for a different project and are not analyzed here. Perceived consensus was assessed on an 8-point scale with categorical response options reflecting specific ranges (e.g., 50–70%); the midpoint of the selected range (e.g., 60%) was used for analysis.

### Results

Separate Type-II ANOVAs for the five dependent variables perceived consensus, AGW acceptance, attribution, trust in climate scientists, and policy support were performed with free-market support as a continuous predictor and the inoculation and misinformation interventions as fully-crossed factors. Table 3 summarizes the means and standard deviations of the dependent variables for each intervention group, whereas Table 4 summarizes the ANOVA results.

**Table 3:** Means (standard deviations) across interventions for Experiment 2.

Dependent Variable	Control	Misinformation-only	Inoculation-only	Inoculation + Misinformatiion
Perceived consensus	54.5 (25.7)	44.5 (30.6)	50.4 (27.6)	51.6 (28.4)
AGW acceptance	3.39 (.72)	3.29 (.97)	3.36 (.79)	3.48 (.74)
Attribution	44.7 (26.2)	40.6 (29.6)	46.3 (29.0)	40.3 (26.1)
Trust in climate scientists	3.06 (.47)	3.12 (.37)	3.03 (.47)	3.02 (.37)
Policy support	3.60 (.75)	3.44 (.92)	3.55 (.81)	3.67 (.67)

**Table 4:** ANOVA results for Experiment 2.

Dependent Variable	Effects	η_p_^2^	F	p
Perceived consensus	Inoculation	.021	.065	.799
	Misinformation	.004	2.85	.092
	Free-Market Support	.102	41.864	< .001***
	Inoculation × Misinformation	.008	3.331	.069
	Inoculation × Free-Market Support	.023	8.217	.004**
	Misinformation × Free-Market Support	.008	2.869	.091
	Inoculation × Misinformation × Free-Market Support	.013	5.198	.023*
AGW acceptance	Inoculation	.019	.371	.543
	Misinformation	.009	.030	.862
	Free-Market Support	.365	218.018	< .001***
	Inoculation × Misinformation	.013	1.098	.295
	Inoculation × Free-Market Support	.022	7.656	.006**
	Misinformation × Free-Market Support	.010	3.549	.060
	Inoculation × Misinformation × Free-Market Support	.017	6.764	.010*
Attribution	Inoculation	.014	.020	.888
	Misinformation	.001	4.440	.036*
	Free-Market Support	.178	82.057	< .001***
	Inoculation × Misinformation	.009	.567	.451
	Inoculation × Free-Market Support	.014	5.112	.024*
	Misinformation × Free-Market Support	.004	1.339	.248
	Inoculation × Misinformation × Free-Market Support	.007	2.957	.086
Trust in climate scientists	Inoculation	.000	2.225	.137
	Misinformation	.005	.426	.514
	Free-Market Support	.004	2.006	.158
	Inoculation × Misinformation	.000	.680	.410
	Inoculation × Free-Market Support	.001	.326	.569
	Misinformation × Free-Market Support	.004	1.666	.198
	Inoculation × Misinformation × Free-Market Support	.001	.309	.579
Policy support	Inoculation	.028	.738	.391
	Misinformation	.005	.203	.653
	Free-Market Support	.310	168.382	< .001***
	Inoculation × Misinformation	.001	2.546	.111
	Inoculation × Free-Market Support	.033	12.829	< .001***
	Misinformation × Free-Market Support	.006	2.227	.136
	Inoculation × Misinformation × Free-Market Support	.002	.727	.394

* p < .05.

** p < .01.

*** p < .001.

**Fig 2:**
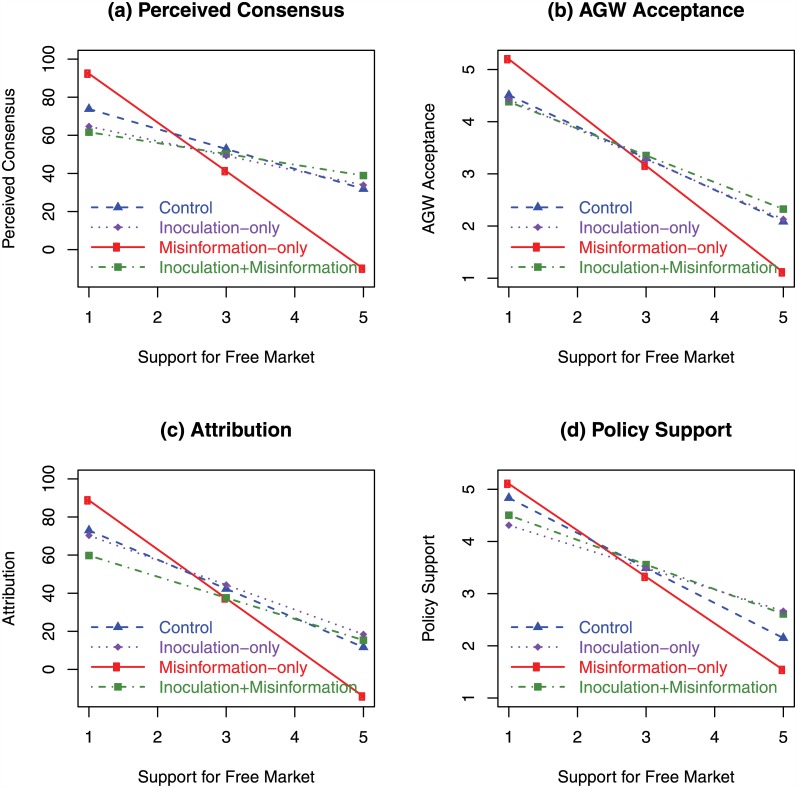
Predicted response in Experiment 2 from linear regression of observed data. Blue dashed line with triangles represents control group, red solid line with circles represents group receiving misinformation-only intervention, purple dotted line with diamonds represents group receiving inoculation-only intervention, green dot-dashed line with squares represents group receiving inoculation plus misinformation. Horizontal axis represents free-market support where 1 corresponds to strong disagreement with unregulated markets and 5 corresponds to strong agreement with unregulated markets. (a) Perceived scientific consensus on AGW. (b) Acceptance of AGW. (c) Attribution of human activity to global warming trends. (d) Support for climate policy.

Fig 2 shows the pattern of interactions between the interventions and free-market support on (a) perceived consensus, (b) AGW acceptance, (c) attribution of human activity, and (d) policy support. Due to the lack of change in trust in scientists across the intervention groups, trust in scientists is not shown in Fig 2. The slopes of the control data (blue dashed lines) show the significant influence of free-market support on all climate attitudes. On average, exposure to the misinformation (red solid lines) had the effect of lowering perceived consensus, AGW acceptance, and attribution, although these differences were not significant. More specifically, misinformation increased polarization, with strong free-market supporters decreasing their climate belief across all four measures.

The inoculation+misinformation group (green dotted lines) showed slightly less polarization than the control group across all four measures, demonstrating that the polarizing influence of misinformation was neutralized by the inoculation. The inoculation-only group (purple, dot-dashed lines) also showed less polarization although our primary interest lies in groups that were exposed to misinformation.

While there was no main effect of inoculation, the two-way interaction between free-market support and the inoculation intervention was significant for perceived consensus, AGW acceptance, attribution, and policy support. The three-way interaction between free-market support, inoculation, and misinformation was significant for perceived consensus and AGW acceptance, and marginally significant for attribution. These three-way interactions indicate that the misinformation had a sizeable effect only if it was not combined with an inoculation, and affected free-market supporters more than participants with low free-market support. In other words, an inoculation was successful in neutralizing the misinformation across all levels of free-market support, and removed the polarizing influence of the misinformation, with the inoculation group showing less polarization than even the control group.

### Discussion

Experiment 2 demonstrated that misinformation—in the form of “fake experts” casting doubt on a scientific consensus—had a polarizing effect on climate attitudes, such that people with low free-market support increased climate acceptance, while people with high free-market support decreased climate acceptance. This form of misinformation may be a contributing factor to the increased polarization on climate change among the U.S. public [60,61]. However, an inoculating message that explains the misinforming technique without mentioning any specifics fully neutralized the polarizing effect of misinformation. This may indicate that when informed of misleading techniques, free-market supporters resist being misled as they see this as a violation of their right to be well-informed.

From a cognitive perspective, it is possible that the inoculation shifted attention from a heuristic surface level to a deeper level of analysis, allowing people to detect patterns of deception [62]. This would imply that inoculation interventions boost strategic monitoring when encoding potential misinformation [63], consistent with the finding that people in a suspicious state are less vulnerable to the influence of misinformation [42]. Experiment 2 thus established the potential utility of general inoculations that explain common misinforming techniques, which can be used to inoculate against different misinforming arguments that employ the same technique.

### Conclusions

Although Experiments 1 and 2 employed different styles of misinformation, both found that inoculation neutralized the negative influence of misinformation on perceived consensus. Experiment 2 also showed that inoculation neutralizes the polarizing influence of misinformation across acceptance of AGW, perceived consensus, and policy support. Our results are consistent with the findings of [33], who observed that combining accurate information with an inoculation explaining the technique underlying the misinformation was effective in neutralizing the misinformation and increasing perceived consensus. The findings from the present study further affirm the effectiveness of inoculation in neutralizing the influence of misinformation.

A number of studies point to possible contributors to the efficacy of inoculation. People in a suspicious state are less influenced by misinformation [42]. The greater influence of inoculation on political conservatives observed in Experiment 2 may be indicative of psychological reactance (a negative reaction to an imposed loss of freedom)[64]. To illustrate, after learning that one has been misinformed, one might perceive the misinformation as an attack on one’s freedom to be accurately informed, which could lead to psychological reactance and a corresponding resistance to the misinformation.

It is also noteworthy that the inoculations in this study did not mention the specific misinformation that was presented after the inoculation, but rather warned about misinformation in a broader sense by explaining the general technique being used to create doubt about an issue in the public’s mind. The purpose of this type of intervention is to stimulate critical thinking through the explanation of argumentative techniques, thus encouraging people to move beyond shallow heuristic-driven processing and engage in deeper, more strategic scrutinizing of the presented information. A consequence of this approach is that generally-framed inoculations could potentially neutralize a number of misleading arguments that employ the same technique or fallacy.

Experiment 1 also found that consensus information was effective in greatly increasing perceived consensus, even in the face of misinformation in the form of false-balance media coverage. The consensus information partially moderated the biasing influence of political ideology, consistent with other studies [29,30]. However, further research is necessary given that this result contrasts with the polarizing influence of consensus information on acceptance of AGW observed with U.S. participants in [12].

The efficacy of consensus information is consistent with other research that has found that perceived scientific consensus is a gateway belief, predicting a variety of climate attitudes including policy support [30]. This dynamic has been recognized by opponents of climate action since the 1990s, who identified manufacturing doubt about the scientific consensus as a key strategy in delaying public support for climate mitigation policies [65,66]. This strategic approach has been documented in an analysis of opinion editorials by conservative columnists from 2007 to 2010, which identified the key climate myths employed [67]. Elsasser and Dunlap observed a highly dismissive stance towards climate science, with the most frequently used argument questioning the existence of a scientific consensus on climate change.

The ongoing focus on questioning the consensus, in concert with the gateway belief status of perceived consensus, underscores the importance of communicating the consensus [68,69]. However, positive consensus messaging is not sufficient, given recent findings that misinformation can undermine positive information about climate change [33,56]. As a complement to positive messages, inoculation interventions are an effective way to neutralize the influence of misinformation.

The research into the effectiveness of inoculating messages is consistent with education research suggesting that teaching approaches directly addressing misconceptions stimulate greater engagement with scientific concepts, which results in more effective and longer-lasting learning [70,71,72,73]. This teaching approach is known as misconception-based learning [74], agnotology-based learning [75], or learning from refutational texts [76]. Misconception-based learning has been successfully implemented in classrooms [77] and a Massive Open Online Course [78]. Further research into inoculation is recommended in order to inform design of more effective misconception-based learning interventions.

## Supporting information

S1 Table Survey items for Experiment 1. Items measuring acceptance of AGW, attribution of human activity, trust, worldview and perceived expertise were averaged within each class of items to calculate the dependent variables.
(DOCX)

S2 Table Survey items for Experiment 2. Items measuring acceptance of AGW, attribution of human activity, trust, worldview and perceived expertise were averaged within each class of items to calculate the dependent variables.
(DOCX)

S1 Text Misinformation intervention text (Experiment 1). Scientists debate causes of climate change.
(DOCX)

S2 Text Consensus-only intervention text (Experiment 1). The Scientific Consensus on Global Warming.
(DOCX)

S3 Text Inoculation-only intervention text (Experiment 1). The Scientific Consensus on Global Warming.
(DOCX)

S4 Text Primer (consensus + inoculation) intervention text (Experiment 1). The Scientific Consensus on Global Warming.
(DOCX)

S5 Text Fact first debriefing text (Experiment 1). (DOCX)

S6 Text Myth first debriefing text (Experiment 1). (DOCX)

S7 Text Fact only debriefing text (Experiment 1). (DOCX)

S8 Text Inoculation intervention text (Experiment 2). Promoting “fake experts” to manufacture doubt about science.
(DOCX)

S9 Text Misinformation intervention text (Experiment 2). The Global Warming Petition Project.
(DOCX)

S10 Text Debriefing text (Experiment 2). Tens of Thousands of “Fake Experts”: Putting the Global Warming Petition Project in proper context.
(DOCX)
