# Supplementary material for: Neutralizing misinformation through inoculation: Exposing misleading argumentation techniques reduces their influence
Source: PLoS One. 2017 May 5;12(5):e0175799. doi: 10.1371/journal.pone.0175799 (PMC5419564; doi:10.1371/journal.pone.0175799)
Supplement: S2 Table — Items measuring acceptance of AGW, attribution of human activity, trust, worldview and perceived expertise were averaged within each class of items to calculate the dependent variables. (DOCX) [file pone.0175799.s002.docx]

**S2 Table: Survey items for Experiment 2.**

Items measuring acceptance of AGW, attribution of human activity, trust, worldview and perceived expertise were averaged within each class of items to calculate the dependent variables.

*Survey items used in Experiment 2*

| Measured Variable | Survey Items |
| --- | --- |
| Gender  (Male, Female) | What sex are you? |
| Age  (18 to 29 years, 30 to 44 years, 45 to 59 years, 60 to 74 years) | How old are you? |
| Income  ($0-$199 per week ($0-$10,399 per year), $200-$599 per week ($10,400-$31,199 per year), $600-$1,249 per week ($31,200-$64,999 per year), $1,250 or more per week ($65,000 or more per year) ) | What is your income? |
| Attention Filter: Primer condition  (Street protests, Fake experts, Flash mobs, Nothing, Physical violence) | What strategy is used by industry groups to manufacture doubt about science? |
| Attention Filter: Misinformation condition  (Less than 100, Between 101 to 1,000, Between 1,001 to 9,000, Between 9,001 to 50,000, More than 50,000) | How many American scientists signed the Global Warming Petition Project? |
| AGW Acceptance (5 item Likert scale from Strongly Disagree to Strongly Agree) | 1. The climate is always changing and what we are currently observing is just natural fluctuation.  2. Most of the warming over the last 50 years is due to the increase in greenhouse gas concentrations.  3. The burning of fossil fuels over the last 50 years has caused serious damage to the planet’s climate.  4. Human CO2 emissions cause climate change.  5. Humans are too insignificant to have an appreciable impact on global temperature. |
| Free Market Support (5 item Likert scale from Strongly Disagree to Strongly Agree) | 1. An economic system based on free markets unrestrained by government interference automatically works best to meet human needs.  2. The free market system may be efficient for resource allocation but it is limited in its capacity to promote social justice.  3. The preservation of the free market system is more important than localized environmental concerns.  4. Free and unregulated markets pose important threats to sustainable development.  5. The free market system is likely to promote unsustainable consumption. |

| Trust in climate scientists (5 item Likert scale from Strongly Disagree to Strongly Agree) | 1. Climate scientists can be depended upon to help increase our understanding of what's happening to our climate.  2. Research that challenges the mainstream point of view is given honest treatment by the scientific community.  3. The process by which scientific papers are peer-reviewed and published is reliable.  4. Climate scientists are sincere in their research into climate.  5. I trust the things that scientists say about climate change. |
| --- | --- |
| Third Person Effect (Not influenced at all, Little influence, Some influence, Moderate influence,  Considerable influence) | To what extent do you think the information about scientific consensus would influence  - you  - your closest friend  - members of your family  - inhabitants of the same State  - other people in general |
| Contribution of human activity to following events (slider from 0 to 100%) | Use the slider to estimate the contribution from human CO2 emissions to cause each event.  1. Increase in atmospheric temperature of 0.8 degrees Celsius since 1880  2. Increase of global sea level of 20cm since 1880  3. Doubling of weather-related natural disasters over last 30 years  4. The 2010 floods in Pakistan  5. Hurricane Sandy in 2012  6. The 2013 Australian heat wave |
| Perceived Scientific Consensus  (Less than 5%, Between 5% to 10%, Between 10% to 30%, Between 30% to 50%, Between 50% to 70%, Between 70% to 90%, Between 90% to 95%, More than 95%) | How many climate experts agree that the global warming we are witnessing is a direct consequence of the burning of fossil fuels by humans? |
| Education  (High school or less, TAFE education, University graduate, Postgraduate work) | What is the highest level of education that you’ve completed and got credit for? |

| Perceived Scientific Consensus (Less than 5%, Between 5% to 10%, Between 10% to 30%, Between 30% to 50%, Between 50% to 70%, Between 70% to 90%, Between 90% to 95%, More than 95%) | How many climate experts agree that the global warming we are witnessing is a direct consequence of the burning of fossil fuels by humans? |
| --- | --- |
| Policy Support (5 point Likert scale from Strongly Support to Strongly Oppose) | 1. Signing an international treaty that requires USA to cut its carbon dioxide emissions by 90% by 2050  2. Signing an international treaty that requires USA to cut its carbon dioxide emissions by 90% by 2050.  3. Adding a surcharge to electrical bills to establish a fund to help make buildings more energy efficient and to teach U.S. citizens how to reduce energy use  4. Requiring electric utilities to produce at least 20% of their electricity from renewable energy sources  5o. Providing tax rebates for people who purchase energy-efficient vehicles or solar panels |
