# Supplementary material for: Neutralizing misinformation through inoculation: Exposing misleading argumentation techniques reduces their influence
Source: PLoS One. 2017 May 5;12(5):e0175799. doi: 10.1371/journal.pone.0175799 (PMC5419564; doi:10.1371/journal.pone.0175799)
Supplement: S1 Text — Scientists debate causes of climate change. (DOCX) [file pone.0175799.s003.docx]

**S1 Text. Misinformation Intervention Text (Experiment 1).**

*Scientists debate causes of climate change*

Scientists have released a controversial new report concluding that humans are causing most of global warming. The report is an assessment of different lines of evidence, including thermometer measurements, satellite data and observations of ocean temperature.

“There are a number of distinctive patterns being observed in our climate,” said climate scientist Dr Jason Griffiths from the University of California, one of the lead authors of the report. “Put together, these patterns strengthen the evidence that humans are causing global warming. They also eliminate other possible natural causes.”

The report lists different lines of evidence supporting human-caused global warming. “Satellites are measuring less heat escaping to space because heat trapping greenhouse gases are absorbing more heat,” said Dr Jones. “Other expected patterns of greenhouse warming have also been observed. For example, winters warming faster than summers is a characteristic pattern of warming from greenhouse gases.”

However, other scientists have criticised the report for not including the views of scientists who disagree with the mainstream position. Dr Bertram Pearse, a climate expert from George Mason University, commented, “This report ignores the fact that eminent climate scientists, who have published hundreds of peer-reviewed papers between them, argue that humans have not had a significant effect on climate. Climate change is still hotly debated among scientists.”

Dr Smith has published studies questioning the link between human activity and climate change. “A key driver of climate is variations in solar activity,” Smith explains. “The sun provides almost all the energy in our climate system. When the sun gets warmer, our planet gets warmer. Over the last few decades, the sun has been unusually warm, achieving its warmest levels in 1,150 years.”
