# Supplementary material for: Neutralizing misinformation through inoculation: Exposing misleading argumentation techniques reduces their influence
Source: PLoS One. 2017 May 5;12(5):e0175799. doi: 10.1371/journal.pone.0175799 (PMC5419564; doi:10.1371/journal.pone.0175799)
Supplement: S2 Text — The Scientific Consensus on Global Warming. (DOCX) [file pone.0175799.s004.docx]

**S2 Text. Consensus-Only Intervention Text (Experiment 1).**

*The Scientific Consensus on Global Warming*

Climate research shows that the temperature of the Earth has been increasing every decade since the 1970s. It is also established that the burning of fossil fuels releases heat-trapping greenhouse gases into the atmosphere. Greenhouse gas emissions have been directly linked to global warming and climate change.  A recent survey of scientists has found that the greater their expertise in climate science, the stronger their agreement on human-caused global warming. Among climate scientists who are actively publishing research into climate, 97 out of 100 agree that global warming is a direct consequence of the burning of fossil fuels by humans.

Overwhelming agreement is also found in published climate research. An analysis of 21 years of climate research found that 97.1% of relevant papers agree with the consensus. Papers rejecting the consensus are a vanishingly small minority.

Surveys of the climate science community and analysis of published climate research both find the same result. Based on the evidence, 97% of climate scientists have concluded that human-caused climate change is happening.
