# Supplementary material for: Neutralizing misinformation through inoculation: Exposing misleading argumentation techniques reduces their influence
Source: PLoS One. 2017 May 5;12(5):e0175799. doi: 10.1371/journal.pone.0175799 (PMC5419564; doi:10.1371/journal.pone.0175799)
Supplement: S4 Text — The Scientific Consensus on Global Warming. (DOCX) [file pone.0175799.s006.docx]

**S4 Text. Primer (Consensus + Inoculation) Intervention Text (Experiment 1)**

*The Scientific Consensus on Global Warming*

Climate research shows that the temperature of the Earth has been increasing every decade since the 1970s. It is also established that the burning of fossil fuels releases heat-trapping greenhouse gases into the atmosphere. Greenhouse gas emissions have been directly linked to global warming and climate change.  A recent survey of scientists has found that the greater their expertise in climate science, the stronger their agreement on human-caused global warming. Among climate scientists who are actively publishing research into climate, 97 out of 100 agree that global warming is a direct consequence of the burning of fossil fuels by humans. Overwhelming agreement is also found in published climate research. An analysis of 21 years of climate research found that 97.1% of relevant papers agree with the consensus. Papers rejecting the consensus are a vanishingly small minority.

Surveys of the climate science community and analysis of published climate research both find the same result. Based on the evidence, 97% of climate scientists have concluded that human-caused climate change is happening. Fossil fuel and political groups try to cast doubt on climate science, in order to delay regulation of the fossil fuel industry. They do this by manufacturing the appearance of an ongoing scientific debate.

The “fake debate” strategy was pioneered by the tobacco industry in the 1970s. They used doctors and scientists to reassure the public that smoking didn’t cause health problems. These spokespeople were either non-experts or among the small minority of scientists who dissented from the scientific consensus that smoking is bad for you. However, in the public’s eye, this conveyed the appearance of ongoing scientific debate.

The “Tobacco Strategy” is being used again, but this time to cast doubt on climate science. Less than 3% of climate scientists disagree with the consensus position that humans are causing global warming. However, when the media present the views of a dissenting scientist alongside a mainstream scientist, the public comes away with the mistaken impression of a 50:50 debate. Ironically, the journalistic standard of giving both sides equal weight has ended up distorting the state of climate science.
