# Supplementary material for: Neutralizing misinformation through inoculation: Exposing misleading argumentation techniques reduces their influence
Source: PLoS One. 2017 May 5;12(5):e0175799. doi: 10.1371/journal.pone.0175799 (PMC5419564; doi:10.1371/journal.pone.0175799)
Supplement: S6 Text — (DOCX) [file pone.0175799.s008.docx]

**S6 Text. Myth First Debriefing Text (Experiment 1)**

Thank you for taking part in today’s study. The session is almost complete but before you finish, it is important that you read the following debriefing information very carefully—you will be assessed on its contents shortly.

Please note that you were exposed to misinformation in this survey. Some of the information in the article “Scientists debate causes of climate change” was in fact misleading and misrepresents the facts.  The aim of the current study is to establish whether it is possible to protect members of the general public from deliberately misleading and inaccurate information—known as “misinformation”.

The following content explains how the previous article misrepresented the facts. Please read the content carefully, as you will be asked questions about it afterwards.

MYTH:  There is no scientific consensus that humans are causing global warming.

FACT: Some groups try to cast doubt on human-caused global warming. They do this by manufacturing the appearance of an ongoing scientific debate. The “fake debate” strategy was pioneered by the tobacco industry in the 1970s. They used doctors and scientists to reassure the public that smoking didn’t cause health problems. These spokespeople were either non-experts or among the small minority of scientists who dissented from the scientific consensus that smoking is bad for you. However, in the public’s eye, this conveyed the appearance of ongoing scientific debate.

The “Tobacco Strategy” is being used again, but this time to cast doubt on climate science. Less than 3% of climate scientists disagree with the consensus position that humans are causing global warming. However, when the media present the views of a dissenting scientist alongside a mainstream scientist, the public comes away with the mistaken impression of a 50:50 debate. Ironically, the journalistic standard of giving both sides equal weight has ended up distorting the state of climate science.

In reality, there is overwhelming agreement among experts that humans are causing global warming. Several surveys of climate scientists have found that 97% agree that humans are causing global warming. Also, over the last 21 years, 97% of scientific papers that state a position on whether humans were causing global warming agree with the consensus. There is overwhelming scientific agreement that humans are driving recent global warming.

MYTH: Recent global warming is caused by the sun, which has been unusually warm.

FACT: A persistent myth is that the Sun is causing global warming. People persist in this myth by cherry picking data. For example, they look at times in the Earth’s past when temperature and solar activity moved in the same direction. But they ignore recent data.

In the last few decades of global warming, solar activity and climate have moved in opposite directions. Surface temperatures have increased and global temperatures hit the hottest on record in 2010. At the same time, the Sun has shown a slight cooling trend. In 2009, solar activity reached its lowest levels in over a century. If anything, the drop in solar activity has had a slight cooling influence on climate in recent decades.

A number of climate patterns confirm that heat trapping greenhouse gases are causing global warming. Winters are warming faster than summers and nights are warming faster than days. The upper atmosphere is cooling while the lower atmosphere warms. These patterns rule out the sun as a potential cause of global warming. They also constitute a ‘fingerprint’ for the fact that humans are causing global warming through greenhouse gas emissions.
