# Supplementary material for: Neutralizing misinformation through inoculation: Exposing misleading argumentation techniques reduces their influence
Source: PLoS One. 2017 May 5;12(5):e0175799. doi: 10.1371/journal.pone.0175799 (PMC5419564; doi:10.1371/journal.pone.0175799)
Supplement: S7 Text — (DOCX) [file pone.0175799.s009.docx]

**S7 Text. Fact Only Debriefing Text (Experiment 1)**

Thank you for taking part in today’s study. The session is almost complete but before you finish, it is important that you read the following debriefing information very carefully—you will be assessed on its contents shortly.

Please note that you were exposed to misinformation in this survey. Some of the information in the article “Scientists debate causes of climate change” was in fact misleading and misrepresents the facts.  The aim of the current study is to establish whether it is possible to protect members of the general public from deliberately misleading and inaccurate information—known as “misinformation”.

The following content explains how the previous article misrepresented the facts. Please read the content carefully, as you will be asked questions about it afterwards.

FACT: 97% of climate scientists agree humans are causing global warming.

As empirical evidence for human-caused global warming accumulated, agreement among the scientific community strengthened. A 2009 survey of Earth scientists found that the greater a scientist’s expertise in climate science, the more likely they were to endorse the consensus. Among actively publishing climate scientists, they found 97% agreement. The same 97% consensus was found in a 2010 analysis of public statements by climate scientists.

An analysis of 21 years of peer-reviewed papers from 1991 to 2011 identified over 4,000 scientific papers that stated a position on whether humans were causing global warming. Among those papers, 97.1% endorsed the consensus. Nearly every reputable, relevant scientific organisation in the world, including the National Academies of Science from 33 different countries, has issued statements endorsing human-caused global warming. There is an overwhelming scientific consensus that humans are causing global warming.

FACT: Climate patterns confirm human-caused global warming.

How do we know that carbon dioxide is the cause of recent global warming? We expect to see a number of distinctive greenhouse patterns in global warming. Observing these patterns strengthens the evidence that humans are causing global warming, as well as eliminating other possible natural causes. Let’s have a look at the many human fingerprints on climate change:   Greenhouse warming is predicted to cause nights to warm faster than days. This is because at night, the Earth’s surface cools by radiating heat out to space. Greenhouse gases trap some of this heat, slowing the night-time cooling. This prediction has been confirmed. Over the last few decades, surface measurements have observed nights warming faster than days. Just as greenhouse gases slow down night-time cooling, they should also slow down winter cooling. Consequently, winters are expected to warm faster than summers. Again, recent analysis of temperature trends over the last few decades bears this out. Both thermometers and satellites find winters warming faster than summers.
