# Supplementary material for: Neutralizing misinformation through inoculation: Exposing misleading argumentation techniques reduces their influence
Source: PLoS One. 2017 May 5;12(5):e0175799. doi: 10.1371/journal.pone.0175799 (PMC5419564; doi:10.1371/journal.pone.0175799)
Supplement: S8 Text — Promoting “fake experts” to manufacture doubt about science. (DOCX) [file pone.0175799.s010.docx]

**S8 Text. Inoculation Intervention Text (Experiment 2)**

*Promoting “fake experts” to manufacture doubt about science*

Sometimes, inconvenient scientific facts threaten the interests of industry groups and organisations. For example, the scientific evidence linking smoking with lung cancer threatened the profits of the tobacco industry. Similarly, scientific evidence linking fossil fuel emissions with global warming threatens the profits of the fossil fuel industry.


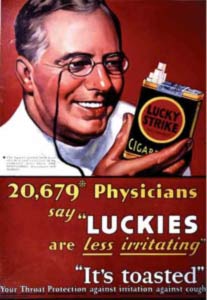
In these cases, a common tactic for industry groups and organisations is to manufacture doubt about the science through the promotion of “fake experts”. Fake experts are spokespeople who convey the impression of expertise in a given area without possessing actual relevant experience. Groups wishing to cast doubt on science often use fake experts to convince the public that the science isn’t settled. Inevitably, the fake expert strategy ends with arguments against government action that threaten the interests of the group.

The tobacco industry pioneered this approach through ad campaigns featuring long lists of doctors endorsing smoking. These ads conveyed the impression that the scientific case linking smoking to lung cancer was not settled. However, the cited “experts” actually consisted of tens of thousands of non-experts. For example, they featured physicians rather than the scientists who actually conducted research into the health impacts of tobacco use. The tobacco industry dubbed this campaign “The Whitecoat Project”, due to the use of spokespeople dressed in white coats to convey the appearance of scientific expertise.

The fake expert strategy is now widely adopted in the climate change arena. To convey the impression that climate scientists are still debating human-caused global warming, opponents of climate action will publish long lists of dissenting scientists. A telling feature of these petitions is promotion of the scientific qualifications of the dissenting scientists but close inspection reveals the scientists do not possess the relevant expertise in climate science.

In other words, these lists consist of fake experts – scientists whose area of expertise is in some domain other than climate change. Drawing upon non-expert opinion on a complex topic such as climate change is equivalent to asking a dentist to perform heart surgery. A white coat alone does not make a heart surgeon.
