# Supplementary material for: Neutralizing misinformation through inoculation: Exposing misleading argumentation techniques reduces their influence
Source: PLoS One. 2017 May 5;12(5):e0175799. doi: 10.1371/journal.pone.0175799 (PMC5419564; doi:10.1371/journal.pone.0175799)
Supplement: S9 Text — The Global Warming Petition Project. (DOCX) [file pone.0175799.s011.docx]

**S9 Text. Misinformation Intervention Text (Experiment 2)**

*The Global Warming Petition Project*

The following text was excerpted from the Global Warming Petition Project website, run by the Oregon Institute for Science & Medicine.

*****

“31,487 American scientists have signed this petition, including 9,029 with PhDs.


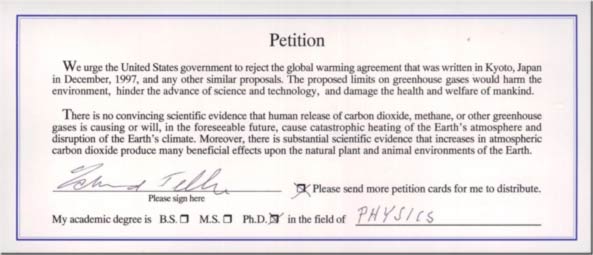


The purpose of the Petition Project is to demonstrate that the claim of “settled science” and an overwhelming “consensus” in favor of the hypothesis of human-caused global warming and consequent climatological damage is wrong. No such consensus or settled science exists. As indicated by the petition text and signatory list, a very large number of American scientists reject this hypothesis.

Publicists at the United Nations, Mr. Al Gore, and their supporters frequently claim that only a few “skeptics” remain – skeptics who are still unconvinced about the existence of a catastrophic human-caused global warming emergency.

It is evident that 31,487 Americans with university degrees in science – including 9,029 PhDs, are not "a few." Moreover, from the clear and strong petition statement that they have signed, it is evident that these 31,487 American scientists are not “skeptics.”

These scientists are instead convinced that the human-caused global warming hypothesis is without scientific validity and that government action on the basis of this hypothesis would unnecessarily and counterproductively damage both human prosperity and the natural environment of the Earth.

The Petition Project was organized by a group of physicists and physical chemists who conduct scientific research at several American scientific institutions. The petition statement and the signatures of its 31,487 signers, however, speak for themselves. The primary relevant role of the organizers is that they are among the 9,029 PhD signers of the petition.”
