# Supplementary material for: Neutralizing misinformation through inoculation: Exposing misleading argumentation techniques reduces their influence
Source: PLoS One. 2017 May 5;12(5):e0175799. doi: 10.1371/journal.pone.0175799 (PMC5419564; doi:10.1371/journal.pone.0175799)
Supplement: S10 Text — Tens of Thousands of “Fake Experts”: Putting the Global Warming Petition Project in proper context. (DOCX) [file pone.0175799.s012.docx]

**S10 Text. Debriefing Text (Experiment 2)**

*Tens of Thousands of “Fake Experts”: Putting the Global Warming Petition Project in proper context*

An earlier page in this survey presented information taken from the Global Warming Petition Project website. This information is highly misleading, designed to manufacture doubt about the scientific consensus that humans are causing global warming.

The scientific consensus on climate change is robust, manifesting in a number of different ways. A strengthening consensus is found in published peer-reviewed research.  There is a consensus among the world’s most prestigious scientific organizations with statements issued by National Academies of Science in 78 countries. There is a consensus among the climate science community with 97% agreement among actively publishing climate scientists.

Given such agreement among an overwhelming majority of climate scientists, a common way to portray a false picture of a divided scientific community is to promote scientists with supposedly impressive credentials who actually possess scant expertise in climate science.

The most prominent example of this “fake experts” strategy is the Petition Project, first published in 2008 by the Oregon Institute of Science and Medicine.  This petition lists over 31,000 scientists who dispute that human activity is disrupting our climate.

With 97% consensus among climate scientists, how is it over 31,000 scientists disagree with the consensus?  This is because around 99.9% of the signatories on the Petition Project are not climate scientists.  Anyone with a Bachelor of Science or higher can be listed.  The list includes graduates of computer science, mechanical engineering, zoology and other fields unrelated to climate science.

The survey also demonstrates a lack of quality control. Characters from the television show M*A*S*H and members of the Spice Girls pop band have been added to the list. In response to this, the Oregon Institute of Science & Medicine commented that there was no way of filtering out fake names from their survey.

Given the lack of climate expertise, the Petition Project is a transparent ploy to foster the impression of ongoing debate on the basic fact of human-caused global warming, among the climate science community where none exists.
